# Designing an automated clinical decision support system to match clinical practice guidelines for opioid therapy for chronic pain

**DOI:** 10.1186/1748-5908-5-26

**Published:** 2010-04-12

**Authors:** Jodie A Trafton, Susana B Martins, Martha C Michel, Dan Wang, Samson W Tu, David J Clark, Jan Elliott, Brigit Vucic, Steve Balt, Michael E Clark, Charles D Sintek, Jack Rosenberg, Denise Daniels, Mary K Goldstein

**Affiliations:** 1 Center for Health Care Evaluation (CHCE), VA Palo Alto Health Care System and Stanford University Medical School, 795 Willow Road (152-MPD), Menlo Park, CA 94025, USA; 2 Geriatrics Research Education and Clinical Center (GRECC), VA Palo Alto Health Care System, 3801 Miranda Ave, Palo Alto, CA 94304-1290, USA; 3 Center for Biomedical Informatics Research, Stanford University Medical School, Medical School Office Building, Room X-215, 251 Campus Drive, Stanford, CA 94305-5479, USA; 4 VA Palo Alto Pain Management Service VA Palo Alto Health Care System and Stanford University Medical School, 3801 Miranda Ave, Palo Alto, CA 94304-1290, USA; 5 Chronic Pain Rehabilitation Program, James A Haley Veterans Hospital, 13000 Bruce B. Downs Blvd., Tampa, FL 33612, USA; 6 VA Eastern Colorado Health Care System, 1055 Clermont Street, Denver, CO 80220, USA; 7 School of Pharmacy, University of Colorado Denver Health Sciences Center, Aurora, CO 80045, USA; 8 Center for Human Development, 4211 Rickey's Way, Suite B, Palo Alto, CA 94306, USA; 9 Center for Primary Care and Outcomes Research (PCOR), Stanford University, 117 Encina Commons, Stanford, CA 94305-6019, USA

## Abstract

**Background:**

Opioid prescribing for chronic pain is common and controversial, but recommended clinical practices are followed inconsistently in many clinical settings. Strategies for increasing adherence to clinical practice guideline recommendations are needed to increase effectiveness and reduce negative consequences of opioid prescribing in chronic pain patients.

**Methods:**

Here we describe the process and outcomes of a project to operationalize the *2003 VA/DOD Clinical Practice Guideline for Opioid Therapy for Chronic Non-Cancer Pain *into a computerized decision support system (DSS) to encourage good opioid prescribing practices during primary care visits. We based the DSS on the existing ATHENA-DSS. We used an iterative process of design, testing, and revision of the DSS by a diverse team including guideline authors, medical informatics experts, clinical content experts, and end-users to convert the written clinical practice guideline into a computable algorithm to generate patient-specific recommendations for care based upon existing information in the electronic medical record (EMR), and a set of clinical tools.

**Results:**

The iterative revision process identified numerous and varied problems with the initially designed system despite diverse expert participation in the design process. The process of operationalizing the guideline identified areas in which the guideline was vague, left decisions to clinical judgment, or required clarification of detail to insure safe clinical implementation. The revisions led to workable solutions to problems, defined the limits of the DSS and its utility in clinical practice, improved integration into clinical workflow, and improved the clarity and accuracy of system recommendations and tools.

**Conclusions:**

Use of this iterative process led to development of a multifunctional DSS that met the approval of the clinical practice guideline authors, content experts, and clinicians involved in testing. The process and experiences described provide a model for development of other DSSs that translate written guidelines into actionable, real-time clinical recommendations.

## Background

Promoting use of good care practices is necessary for safe and effective use of opioid therapy for chronic non-cancer pain, but achieving provider adherence to clinical practice guideline (CPG) recommended care practices has proven difficult in most primary health care settings [1-3]. Increased attention to the importance of pain management has led to increased prescribing of analgesic medications [4]. Opioid analgesics are among the most prescribed medications in the US today [5,6] and, as of 2008, hydrocodone was the top prescribed medication in the country [4]. However, increased use of these powerful and potentially addictive medications has had negative consequences. Rates of opioid overdose, prescription opioid misuse and addiction, diversion of prescribed medications toward illicit use, and opioid-related legal suits against physicians have all increased to a disturbing extent [4,5]. Use of recommended care practices is considered essential for minimizing these negative consequences without reversing gains made in improving pain management in clinical settings.

In 2003, the Veterans Administration (VA)/Department of Defense (DOD) published a CPG for use of opioid therapy for the treatment of chronic non-cancer pain [7]. The goals included using evidence-based recommendations to improve analgesia, promote uniformity of care, and decrease related morbidity of patients with non-cancer chronic pain in the primary care setting. This guideline provides detailed information about appropriate dosing, including protocols for initiation, titration, and cessation of the most commonly used opioid medications. It provides information on potential contraindications for opioid therapy for chronic pain and suggestions for opioid management in patients at higher risk of misuse, diversion, adverse effects, overdose, and/or lack of efficacy. A substantial portion of the guideline focuses on processes of care. For example, the guideline encourages clinicians to: regularly conduct assessments of pain and functioning; use urine drug screening protocols to discourage and detect medication misuse and diversion; obtain written agreement on the parameters and responsibilities of the patient regarding the opioid prescription; provide clear education on both the risks and realistic level of benefit from opioid analgesics; and carefully document and follow treatment plans. This framework can increase clinician's confidence in appropriately prescribing opioid therapy.

Despite expert consensus on the importance of adherence to these care guidelines, there is little evidence that they are consistently followed in actual clinical practice [8]. Numerous barriers to providing guideline-adherent care exist [9]. Clinicians report lack of training in both pain management and addiction medicine and are uncomfortable assessing and treating these conditions. Moreover, patient-provider communication about opioids is complicated by: the subjective nature of pain experience, which prevents physicians from objectively verifying the severity of the pain condition; the reinforcing effects of opioid drugs, which may lead to either deliberate or unknowing attempts by the patient to obtain opioid medications; provider and patient fears about the consequences of either prescribing a potentially addictive medication or under-managing pain; and stigma associated with substance use disorders [10-12]. Because of these communication difficulties, providers may be hesitant to prescribe opioid medications initially, to discontinue medication when there is no clear sign of benefit, and to address the addictive nature of opioid analgesics and the possibility of misuse. In all cases, these behaviors lead to suboptimal care. Additionally, poor care coordination within the health care system contributes to poor opioid management [13]. Lack of clear documentation of pain management plans and opioid use agreements and lack of communication between providers can lead to inconsistent treatment and poor prescribing decisions that contribute to misuse and poor pain management. Lastly, good care practices take time, and time limitations and competing demands during outpatient visits in primary care may limit clinician adherence to guidelines.

Developing health services interventions that address these barriers is essential for improving opioid management in chronic pain. A computerized decision support system (DSS) may provide such an intervention [14,15], and some DSSs have been shown to increase adherence to guideline recommended care [16]. Hunt and colleagues systematically reviewed randomized controlled trials of DSSs, defined as 'any electronic or non-electronic system designed to aid directly in clinical decision making, in which characteristics of individual patients are used to generate patient-specific assessments or recommendations that are then presented to clinicians for consideration' [17]. Kawamoto and colleagues identified features that were independently associated with improved clinical practice in a multiple regression analysis. These included: automatic delivery, presentation of the DSS when and where clinical decision making occurs, provision of concrete recommendations of how to proceed, and computer-based generation of decision support [18]. A model computerized DSS (ATHENA-DSS) that links with the electronic medical record (EMR) system used by the VA Health Care System (VistA) was designed to provide these key features [19-22]. ATHENA-DSS, developed using the EON guideline decision-support technology [23,24], accesses patient information in the EMR, evaluates this information in terms of a knowledge base consisting of encoded CPG recommendations, generates patient-specific recommendations, and presents a graphical user interface with these recommendations along with tools and information support to clinicians when they open the EMR of a relevant patient at the time of the clinic visit.

**Figure 1:**
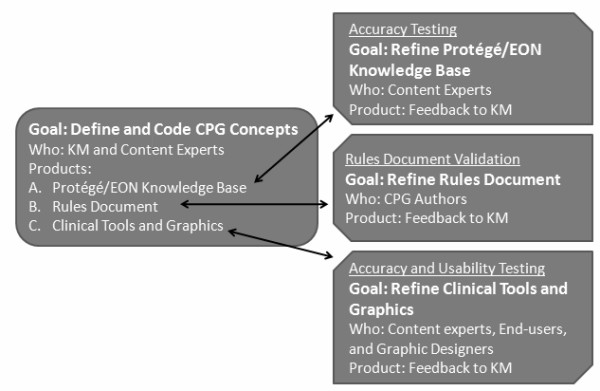
**Model of CPG translation and revision**. This figure describes the products, review processes and reviewers for the three main products of the ATHENA-OT CPG translation project.

We used an iterative development process involving authors of the CPG, local content experts, end-users (*i.e.*, opioid prescribers), knowledge modelers, graphic designers, and systems software engineers to modify the initial ATHENA-DSS, ATHENA-Hypertension (HTN), to guide evidence-based opioid prescribing (Figure 1). We named this newly developed system ATHENA-Opioid Therapy (ATHENA-OT) [25]. Here, we describe the process and outcome of this iterative development via which we operationalized CPG information into a computer-interpretable knowledge base to provide patient-specific recommendations for care, and clinical tools to encourage good care practices in opioid prescribing. Iterative usability testing was also a crucial component of ATHENA-OT development, but these processes will be described elsewhere [26]. A valuable part of our process is collaboration of the DSS developers directly with the CPG authors. The process described provides a model for translating guidelines into DSSs, including methods to ensure that the DSS retains the intent of the CPG authors and encourages use of good care practices through inclusion of patient-specific recommendations and clinical tools.

## Methods

The patient safety features and a thorough description of the ATHENA-OT graphical user interface have been described previously [25]. This study was approved and overseen by the Stanford University Human Research Protection Program and the VA Palo Alto Health Care System Research and Development Committee.

In the process described, the team started with the CPG and translated it into three primary products: an operationalized algorithm in Protégé/EON, a matching written Rules Document, plus a set of clinical tools (Figure 1). We based our guideline translation process on experience gained in development of ATHENA-Hypertension as well as general principles from medical informatics literature encouraging iterative design based on interim evaluation and testing (for example, the ADDIE (Analysis, Design, Development, Implementation and Evaluation) process [27]. The accuracy testing procedures for both the Protégé/EON algorithm and the clinical tools were adapted from those initially designed and successfully used by the ATHENA-Hypertension development team [28]. The Rules Document validation process was designed for ATHENA-OT and has not been previously described, and thus we report this process and findings in greater detail.

A knowledge management team (KM) consisting of the study managers, knowledge modelers (SBM and MM, medical informaticists with expertise in translation of clinical knowledge into encoded computer-interpretable formats using a knowledge acquisition program called Protégé [29]), and system software experts drafted, revised, and managed the review of these 3 products. Each of these three products were reviewed and revised through separate procedures and distinct, but overlapping teams. Revisions to the Protégé/EON algorithm and the Rules Document were made in tandem to maintain consistency, based on feedback from the accuracy and rules validation testing. These processes occurred iteratively during ATHENA-OT development. Each of the processes, as well as major revisions, are described below.

### Drafting a Rules Document and operationalized algorithm in Protégé/EON

To create an encoded guideline, one must specify details that are not explicitly included in the CPG [30]. For example, the CPG for opioid therapy states: 'long-acting agents are effective for continuous, chronic pain'. This statement fails to specify which medications should be considered 'long-acting agents' and the definition of continuous, chronic pain. In order for the computer to be able to use this information, the definitions of 'long-acting agents' and 'continuous, chronic pain' must be explicitly defined or operationalized.

To operationalize the 2003 VA/DOD 'Clinical Practice Guideline for Opioid Therapy for Chronic Non-Cancer Pain', the KM, the medical director, and clinical nurse specialist who direct the VA Palo Alto Health Care System Pain Management Clinic worked collaboratively to create a draft of the guideline knowledge to be encoded in Protégé and specify concepts that were not clearly defined. The process involved the KM reviewing the CPG and attempting to translate the contained recommendations into well-defined concepts that could be encoded in terms of a computer-interpretable model of CPGs [23]. The KM referred questions to clinical experts to iteratively refine the encoded guideline. In addition to encoding the knowledge in Protégé/EON, a 'Rules Document' was created that provided a written description in simple but highly-specified English of the included concepts and rules that the developers intended to encode. The Rules Document serves as a format for review by clinicians and CPG authors [28].

### Review processes

#### Accuracy testing of the Protégé/EON algorithm

Experts in opioid therapy for chronic pain, including the clinical nurse specialist at the VA Palo Alto Pain Management Clinic, a Ph.D. researcher specializing in opioid pharmacology and behavior, a primary-care physician, and a psychiatrist, pilot tested the encoded guideline iteratively during the development and refinement of the operationalized algorithm. Accuracy testing involved examination of ATHENA-OT recommendations for real patient cases with recent primary care visits selected from the VA Palo Alto's EMR. ATHENA-OT generated definitions and recommendations that were compared to information in the EMR and to expert assessment of the patient case in the EMR using the CPG recommendations. Straightforward errors in the generated recommendations were noted and sent to the KM for immediate correction (*e.g.*, miscoding of a diagnosis or minor wording changes). Concerns involving clinical recommendations were first discussed by the expert reviewers, and final suggestions for changes to the encoded guideline were sent to the KM. When suggestions were outside the boundaries of the DSS, the KM met again with the expert reviewers to discuss options and insure that the boundaries were made clear to clinical users to avoid false expectations on the part of the user about the system's capabilities.

#### Validation of the draft Rules Document by authors of the CPG

Once the encoded guideline had been pilot tested for accuracy and the Rules Document updated to match the current content of the encoded guideline, the Rules Document was sent to three authors of the 2003 VA/DOD *Opioid Therapy for Chronic Non-Cancer Pain *CPG (MC, JR, CS). For each clinical rule, the authors were asked to consider the CPG and indicate first whether the clinical rule agreed with the intent of the CPG as written or was incorrect based upon the intent of the CPG. Second, they were asked to comment when the Rules Document was not clear and further clarification of intent of the encoded guideline was required. The guideline-authors' comments included details regarding clinical rules with which they disagreed or that they thought needed refinement. This feedback was used to revise the Rules Document and Protégé/EON algorithm to address the guideline authors' concerns.

#### Clinical tool design

The CPG contained many recommendations to support good clinical care practices that were best shared with primary care clinicians through easily accessible tools (links within the DSS). In discussion with VA Palo Alto clinicians in the Pain Management and Primary Care Clinics, the KM developed information sheets and other clinical tools within ATHENA-OT to facilitate adherence to the CPG recommendations. These tools were vetted and, where appropriate, pilot tested for accuracy by the clinical staff at the pain management clinic and opioid experts on the project team.

#### User interface design

A final step in translating the CPG into ATHENA-OT was determining how to present patient-specific recommendations and clinical tools to the clinicians most effectively. Accordingly, in consultation with a graphic design firm, we used an iterative design and evaluation process to optimize the graphical user interface. This process is described elsewhere [26]. While it is difficult to completely dissociate the development of the user interface from the process of translating the guideline, here we focus only on development of clinical tools and patient-specific recommendations suggested in the CPG.

#### Revision of the Rules Document and Protégé/EON algorithm

Based upon feedback from the review processes, a substantial redesign of the algorithm was conducted. Following system redesign, in depth re-testing of the accuracy the Protégé/EON algorithm was conducted, and the revised Rules Document was again sent out to the three CPG authors for a second round of validation. The CPG authors indicated additional areas of disagreement or requirements for clarification. In this round, the exact wording of DSS recommendations was provided for review. Final consensus on the Rules Document was obtained by conducting follow-up phone calls and emails with the CPG authors where remaining changes were planned, specified, and approved.

## Results

### Drafting of a Rules Document and operational algorithm in Protégé/EON

The KM and clinical experts met approximately 30 times over the course of nine months, and had extensive email communication. The encoded guideline in Protégé/EON included: operationalized definitions of all the concepts included in the guideline (*e.g.*, the ICD-9 codes corresponding to a named diagnosis, or the pharmacy codes for medications of a specified class); an algorithm that operationalized guideline recommendations in terms of the relevant patient scenarios, management decisions, and alternative actions; a collection of situations that warrant warning messages; and declarative specification of the indications, contraindications, and dose ranges of classes of opioids.

Because it is not possible to encode all medical knowledge, clinical DSSs must attend to specifying boundaries and planning system performance at the boundaries [31,32]. Some of the guideline knowledge relies on clinical concepts that are difficult to operationalize and/or call for data not available in computable formats from the patients' EMR. We set these as boundaries of ATHENA-OT and specified plans for system behavior at the boundary (see Table 1 for examples).

**Table 1:** Examples of Boundaries of ATHENA-Opioid Therapy

Issue	Solution
Lack of expert consensus on specific criteria for judging an opioid trial as failed and thus appropriate to discontinue.	The determination of whether to discontinue an opioid medication was left to clinical judgment and always presented as an option. Detailed instructions on how but not when to discontinue the opioid medication were provided.

CPG was written to guide prescribing for non-cancer pain. Some patients have cancer plus pain from non-cancer-related causes, making it unclear whether the CPG was appropriate to apply.	ATHENA-OT issued a warning when the patient had cancer and indicated that system recommendations may not be appropriate if the patient's pain was caused by the cancer.

Determination of the severity of illness requires clinical assessment during the current visit.	ATHENA-OT issued a warning about potentially concerning diagnoses and recommended that the clinician assess the patient's current status to clarify if opioid dose adjustments were necessary.

In the electronic medical record (VistA), allergies are not distinguished from adverse events.	As a conservative measure, any record of an allergy/adverse event was considered an allergy, and recommendations were generated based on this assumption. This definition was clarified in clinician training sessions.

The table above provides examples of portions of the guideline that were not encoded. For each example, we describe how this boundary of the DSS was indicated in ATHENA-OT.

### Round one review

#### Accuracy testing of the Protégé/EON algorithm

Accuracy testing of the Protégé/EON algorithm identified numerous errors in CPG coding that were subsequently corrected. Commonly identified technical errors included omissions of important medical record data in the ATHENA-OT data extract and miscoding of concepts such that recommendations were not produced as planned. Less commonly, clinical cases that had not been anticipated previously by the KM and clinical experts were identified that required refinement of recommendations to align with the assumed intent of the CPG.

In round one assessment the CPG authors agreed with many but not all the clinical rules specified in the Rules Document (table 2). However, they also identified some broad conceptual problems with the design of the DSS. CPG authors often objected to strict recommendations based on patient diagnoses. For example, the initial DSS eligibility criteria excluded all patients with a cancer diagnosis because the guideline indicated that the recommendations for opioid therapy were specifically for non-cancer pain. CPG authors highlighted their disagreement with this decision because it would prevent the system from providing recommendations to those patients with non-cancer-related chronic pain who also happened to have cancer. There was also some disagreement among CPG authors about the broad issue of whether ATHENA-OT should provide firm discontinuation recommendations based on the presence of substance abuse and psychiatric diagnosis. Moreover, comments from CPG authors made it clear that accurate decisions about whether medication should be increased, decreased, or discontinued could not be made using only information available in the EMR. These comments helped clarify situations where clinicians might appropriately either ignore or decide against guideline recommended actions based on information not in the EMR, allowing alteration of the DSS to encourage less rigid use of recommendations in these circumstances.

**Table 2:** Clinical practice guideline author agreement with Rules Document

Rule Category	Agreement (%)		Clarification (%)	
	**Round one**	**Round two**	**Round one**	**Round two**

**Drug recommendations overall**	82	79	24	21

1) Initiation dosing	93	43	7	64

2) Titration dosing	89	89	11	0

3) Switching dosing	100	100	14	0

4) Cessation dosing	28	100	86	0

5) Medication choice	-	100	-	0

**Contraindications/warnings overall**	84	86	44	12

1) Medical contraindications	80	69	75	0

2) Psychiatric contraindications	94	89	30	15

3) Psychosocial contraindications	63	100	47	0

Patient eligibility and exclusion	66	100	44	0

% Agreement indicates the percentage of rules in each category for which all three authors indicated agreement. % Clarification indicates the percentage of rules in each category where at least one author identified problems with the rule that needed to be addressed to ensure agreement with the CPG.

Thus, CPG authors' comments in round one Rules Document assessment suggested problems with an overall decision support strategy of providing clinicians with a single actionable recommendation for opioid prescribing (*e.g.*, 'increase dose of medication [X] by [Y] mg'). Guideline author comments made it clear that clinician judgment, patient preferences, and information not available in the EMR were crucial to providing CPG-adherent opioid therapy, and that a decision support strategy providing greater clinical flexibility would be more appropriate.

#### Revision of the Rules Document and Protégé/EON algorithm

A substantial redesign was conducted. This redesign addressed several concerns that had not previously been solved because of lack of consensus or detail in the CPG or lack of information in the EMR. Instead of displaying our best 'guess' about the recommended course for opioid prescribing, we decided to display all possible therapeutic options for the provider to select from based on clinical judgment. Specifically, we switched from presenting clinicians with detailed procedural or dosing recommendations for the system's one best guess regarding the appropriate strategy for dosing change (*i.e.*, start medication, increase dose, decrease dose, switch to a different medication, or stop medication) to providing detailed procedural or dosing recommendations for all possible options with presentation of indications and contraindications for each choice. This modification emphasized the fact that clinical decisions about overall strategy for opioid therapy require assessment of physical and social functioning and the patients' goals and preferences for treatment as well as clinical judgement. Thus, this clinical decision requires clinician-patient discussion during the visit and cannot be made based on information solely in the EMR. This design choice allowed the team to focus ATHENA-OT on insuring safe and informed implementation of treatment strategies following a shared clinical decision-making model [11].

#### Round two review of the Protégé/EON algorithm and Rules Document

Accuracy testing was conducted again. Errors in Protégé/EON coding were identified and corrected, and wording of recommendations was edited as recommended by the expert testers. After the Rules Document was updated based on the comments from the initial assessment and the system redesign, CPG authors re-evaluated the clinical rules (See Appendix 2 for the Rules Document for round two review). Notably, in round two, the wording of clinical recommendations was included in the Rules Document for approval and comment. In this second round review, CPG authors indicated increased consensus on the Rules Document used for ATHENA-OT. The CPG authors agreed with a higher proportion of the clinical rules as written, and they requested clarification of fewer clinical rules, with the notable exception of several suggestions for detailed modifications which affected many of the rules for initiation dosing and contraindication warnings (Table 2). These exceptions are discussed below. As the Rules Document became more defined and refined, CPG author concerns and comments became more detailed. Suggested changes and clarifications became more minor, although the number of suggestions did not decline in every category. While major redesign of ATHENA-OT was required to address guideline author comments in round one, round two revisions were minor enough to be resolved with small wording changes in existing patient-specific recommendations or slight modification of diagnostic definitions. These changes were discussed and approved in follow-up telephone calls with the CPG authors.

Table 3 shows example areas of disagreement of the CPG authors with the Rules Document, and the revisions made in response. One disagreement that affected a cluster of recommendations was not identified until round two, but was important for patient safety and included details not explicitly specified in the CPG. Specifically, a guideline author expressed concern about non-steroidal anti-inflammatory drug (NSAID) or acetaminophen overdose related to prescription of short-acting opioid medications combined with NSAIDs/acetaminophen in the absence of an assessment of additional use of over-the-counter or prescribed NSAIDs/acetaminophen, and suggested this be noted in ATHENA-OT initiation recommendations. We note that this concern was identified only very vaguely in the CPG, which stated just that an assessment of patient's current medications be conducted before initiation of opioid therapy. This omission affected many of the recommendations for initiation of short-acting medication, but was easily corrected through a simple change of wording in the recommendations. Additionally, once the accuracy of clinical recommendations was less of an issue, CPG authors began to consider the relative importance of recommendations in their decisions. This revealed differences of opinion about the strength of wording of some recommendations, and whether accurate, but rarely important, recommendations should be displayed at all. For example, we issued a warning message about use of opioid therapy in patients with a diagnosis of a DSM-IV Axis II personality disorder, and a guideline author suggested the message be specific to psychopathy, sociopathy, anti-social personality, and borderline personality. These requests for changes during second round evaluation led to lower agreement rates for initiation dosing, and medical and psychiatric contraindications (Table 3). However, the CPG authors did not find their colleagues' suggested revisions controversial, and there was general agreement during follow-up that addition of these details to recommendations beyond the level of detail in the original CPG represented improvements.

**Table 3:** Examples of areas of disagreement in the Rules Document and revisions

Round	Examples of disagreement	Guideline author's reasoning	Revisions made in response
Round one	Strict discontinuation messages based on substance abuse or psychiatric diagnosis	Need to evaluate current status of diagnosis	Updated algorithm to generate all therapeutic options with contraindications for provider to consider and apply based on clinical judgement

Round one	Warning for patients that live >200 miles from VA.	Patients may have continuity of care even if living far away.	Recommendation to not provide opioid therapy and refer patient for care with a local provider was removed

Round two	Dose recommendation for short acting opioids combined with NSAIDs/acetaminophen	Concern about dose of NSAIDs/acetaminophen with medication combinations	Wording of message updated

Round two	Warning about patients with personality disorder	Warn specifically about anti-social and borderline personality disorders	Restricted warnings to persons with these specific personality disorder diagnoses.

#### Clinical tools development

The CPG provided information regarding definitions, assessment and documentation requirements, patient education materials, clinical referral needs, and dosing conversion tables that could not be efficiently presented as patient-specific recommendations. To include these elements of the guideline in ATHENA-OT, we developed clinical tools and information sheets that were incorporated into menus on the graphical user interface. These tools were derived from the CPG with additional input from pain experts and primary care providers.

These clinical tools included interactive systems, such as a conversion calculator (Additional file 1) that was created based on tables in the guideline to facilitate dose conversion when switching between opioids. Interactive assessment instruments were also developed to improve CPG adherence. The CPG recommends that clinicians conduct and document a comprehensive biopsychosocial assessment of pain before opioid prescribing and at subsequent visits. Discussion with local primary care clinicians in usability testing revealed that such assessments were not completed in a standardized manner and that clinicians were uncertain about the detailed elements that should be included in their assessments. Experts and target users agreed that a standardized pain assessment form that could be written back into the EMR would be helpful for clinicians conducting these pain assessments and reassessments. We designed such a pain assessment (Additional file 2) based upon existing tools and the recommendations of the local pain clinic staff. This tool provided check boxes to record patient information that could be written back into a structured progress note (Additional file 3) in the patient EMR for clinician review and signature.

Some information sheets were created to provide locally tailored versions of information for providing guideline-adherent therapy. For example, a contact list for local referral sources for pain, addiction, rehabilitation, and behavioral therapy was created. Similarly, state legal requirements for documentation of opioid management were outlined in another information sheet. We also included simple pre-existing information sheets and patient education documents to ensure that these were readily accessible to clinicians.

## Discussion

Collaboration on an iterative design process between the ATHENA-OT developers, local content experts, and the authors of the CPG for management of opioid therapy for chronic pain identified issues that arise in encoding a DSS and provided a mechanism for their resolution, resulting in an automated DSS that fulfils the intentions of the CPG authors.

Clinicians who provide care for patients based on guidelines must operationalize them to carry out care. Operationalizing CPGs for automated DSS highlights the context, assumptions, ambiguities, and gaps that are inherent in the usual formats of CPGs [30]. Encoding the DSS requires interpreting the context, specifying concepts, clarifying embedded assumptions, spanning gaps, and resolving ambiguities in source documents. Evidence sources for recommendations can sometimes serve as sources for specification of concepts (*e.g.*, which specific diagnoses were included in the original study that forms the evidence base for a particular recommendation) or target population assumptions, but do not fill all the gaps. Consensus-based recommendations may present a particular challenge for translation into computerized DSS. These recommendations typically do not reference scientific studies on which definitions of concepts or cut-points for decisions could be based [30]. Without participation of the CPG authors, who have extensive expert knowledge beyond what is written into the CPG document, the intent or specificity of the guideline may be altered as it is translated into a computerized DSS. In developing ATHENA-OT, we used a two-phase process including accuracy testing plus a review of the guideline rules by three authors of the CPG, followed by a final round of telephone and email communications to arrive at a final version meeting approval of the CPG authors.

This process clarified details of the recommendations and better specified patient populations for whom recommendations were relevant. However, the process was made more challenging by the consensus nature of the guidelines. There was not perfect agreement among the CPG authors for all recommendations. Moreover, in some cases, it seemed apparent that some vague areas of the guideline were purposefully written to be vague, an option that could not be directly incorporated in the computerized algorithm beyond careful wording of the recommendations. Encoding the guideline into a DSS revealed areas of the guideline that lacked specificity and therefore might be difficult to implement both in the DSS and clinically. We note that the opioid therapy guidelines may have been more challenging to operationalize than other guidelines in this regard, due to the extreme heterogeneity of patients and underlying health conditions, limited evidence-base for many criteria, and controversies surrounding treatment.

Operationalizing the opioid therapy for chronic pain guideline was further complicated by the fact that good opioid prescribing decisions require consideration of behavioral, mental health, and psychosocial conditions and functioning. Moreover, patient preferences and goals necessarily influence decisions about use of opioid therapy. Prescribing decisions involve striking a balance between pain control, adverse-effect management, physical and emotional function, and addiction risk. This information can be efficiently obtained only in discussion between patient and provider; there is little to no computable information recorded in the medical record to indicate biopsychosocial functioning and patient preferences to guide such decisions. What information is available in the medical record is typically contained in free-text notes rather than structured data fields and would require advanced text mining algorithms to access, an option that may be available in the future. To design a functional DSS, the expert team was required to consider how the algorithm could usefully support prescribing decisions while not having access to crucial information required to guide opioid prescribing. To overcome these limitations, the expert team designed a DSS that would highlight important clinical information available in the medical record, provide many tools to support shared decision making, facilitate appropriate documentation, and present a checklist of important items to review when considering opioid therapy.

An issue that led to difficulties in getting expert consensus was the problem of differentiating between accurate information and information that was of high clinical priority. There was disagreement among the expert team and CPG authors about whether all accurate information should be provided by the DSS, or whether messages should be limited to issues that were clearly important enough to warrant taking primary care clinicians' time. The process of obtaining consensus on the system could be streamlined by clarifying whether the goal of rules development was to identify all clinically accurate recommendations that could be made from EMR data, or to identify important clinical recommendations for typical primary care practice. The process of analysing the encoded guideline rules presented the guideline information in a different way to guideline authors leading to new insight on their part. This insight made explicit some implicit assumptions by showing varying ways the guideline could be interpreted and applied, which may not have been the intention of the guideline authors.

The wording of recommendations and design of clinical tools evolved alongside the operationalization for the guideline. Striking a balance between detail, accuracy, and clinical utility was difficult, with disagreements among the diverse members of the development and evaluation team leading to dynamic changes in the content of recommendations in terms of prioritization, tone, and wordiness. The CPG suggested tools that would facilitate guideline recommended care processes, but expert and end-user input was needed to optimize content and design. For example, based on feedback from clinician members of the team and usability testing [26], we implemented a write back capacity of the pain assessment template.

Experience with this iterative process suggested several improvements that might have streamlined CPG translation into a DSS. Specifically, we would now recommend:

1. Including an explicit focus on defining the boundaries or limits of the DSS and how they would be handled at the start of the translation process. Outlining these in the Rules Document for review would help ensure that they are considered and addressed thoroughly during initial DSS design.

2. Collecting information from CPG authors regarding prioritization of DSS recommendations during review of the Rules Document. Both CPG authors and end-users participating in usability testing [31] brought up issues regarding distinguishing the clinical importance of recommendations.

3. Presenting specific wording of DSS recommendations as well as an indication of whether the wording should be presented on the main screen or only after end-user interaction with the system (*e.g.*, presented following a mouse click) to CPG authors to review as part of the Rules Document. Subtle wording changes in the DSS recommendations could alter author consensus on recommendations.

## Summary

An iterative process of drafting, testing, reviewing, and revising the DSS content enabled us to develop a DSS that usefully operationalized the written CPG for opioid therapy for chronic pain into a system that could provide real-time decision support in clinical settings. Including guideline authors alongside the local expert team in the iterative development of the content resulted in a product that was considered consistent with the intent of the guideline and amenable to implementation with the local EMR and patient population. The process and experiences described here provide a model for development of the content other DSSs attempting to translate written CPGs into actionable, real-time clinical recommendations and tools.

## Competing interests

The authors declare that they have no competing interests, with the exception of Charles Sintek who declares he is currently on the speaker's bureau for Ortho-McNeil Pharmaceutical (markets Nucynta^®^) and was on the speaker's bureau for Organon Pharmaceutical (markets morphine CR (Avinza^®^)) in the past five years. The ATHENA-OT developers plan to make it available for open-source use in the future; there are no plans for commercial licensing so there is no financial conflict of interest.

## Authors' contributions

JT supervised and provided final approval of iterative revisions of ATHENA-Opioid Therapy content and presentation, tested ATHENA-Opioid Therapy, assisted with data analysis, and drafted the manuscript. MM and SM developed the operationalized clinical algorithm, identified problems requiring alteration, implemented agreed upon revisions, conducted data analysis, and assisted with manuscript drafting. SWT developed the EON guideline-encoding format and the execution engine, provided suggestions for content presentation, and assisted with guideline encoding. DC and JE developed and approved initial criteria for operationalization of guideline concepts and tested ATHENA-Opioid Therapy. BV created specifications for development of ATHENA Opioid Therapy. SB tested ATHENA-Opioid Therapy and made suggestions for revisions. MC, CS, and JR analysed the Rules Document for concordance with the written CPG and made suggestions for revisions. DW revised and upgraded the original ATHENA-DSS software for this application, implemented suggested interface components, enabled deployment of the system, made suggestions for revisions, and extracted patient data. MKG supervised development of the original ATHENA-DSS and provided guidance and recommendations for modification of the system for this clinical application, participated in planning the original proposal, and participated in early setup of the ATHENA-OT project. DD conceived of the study, wrote the original grant proposal, and supervised initial design of the ATHENA-Opioid Therapy DSS. All authors read, provided suggestions for revising, and approved the final manuscript.

## Supplementary Material

Additional file 1 **Conversion calculator tool**. Picture of a conversion calculator tool to support clinicians calculating dose equivalents when converting from one opioid drug to another. Click here for file

Additional file 2 **Pain Assessment tool**. Picture of the pain assessment tool clinicians can interact with and write to the medical record. Click here for file

Additional file 3 **Note in medical record from pain assessment tool**. This is a picture of a note written to the electronic medical record when clinicians complete the pain assessment tool. Click here for file
